# The mutual effect of pre-pregnancy body mass index, waist circumference and gestational weight gain on obesity-related adverse pregnancy outcomes: A birth cohort study

**DOI:** 10.1371/journal.pone.0177418

**Published:** 2017-06-02

**Authors:** Xiao Gao, Yan Yan, Shiting Xiang, Guangyu Zeng, Shiping Liu, Tingting Sha, Qiong He, Hongyan Li, Shan Tan, Cheng Chen, Ling Li, Qiang Yan

**Affiliations:** 1 Department of Epidemiology and Health Statistics, Xiangya School of Public Health, Central South University, Changsha, Hunan Province, China; 2 Department of Maternal and Child Health, Kaifu District Health Bureau, Changsha, China; Universidade de Sao Paulo, BRAZIL

## Abstract

**Objective:**

The aim of this study was to examine the mutual effect of pre-pregnancy body mass index (BMI), waist circumference (WC) and gestational weight gain (GWG) on obesity-related adverse pregnancy outcomes.

**Methods:**

This birth cohort study was conducted in three Streets in Changsha, China, including a total of 976 mother-child pairs. All data was collected within 15 days after deliveries from a self-administered questionnaire, maternal health manual and perinatal health care information system. Multivariate logistic regression models were conducted to estimate the effects of maternal pre-pregnancy BMI, WC and GWG on obesity-related adverse pregnancy outcomes including gestational diabetes mellitus (GDM), primary cesarean section (P-CS), large for gestational age (LGA) and composite outcome (one or more adverse pregnancy outcomes)

**Results:**

After controlling for all confounders, both maternal pre-pregnancy overweight/obesity and central adiposity contributed to increased risks of GDM [ORs 95% CIs = 2.19 (1.02–4.76) and 2.26 (1.11–4.60), respectively], P-CS [ORs 95% CIs = 1.66 (1.05–2.65) and 1.71 (1.11–2.63), respectively], LGA [ORs 95% CIs = 1.93 (1.07–3.50) and 2.14 (1.21–3.75), respectively] and composite outcome [ORs 95% CIs = 1.82 (1.15–2.87) and 1.98 (1.30–3.01), respectively] compared with mothers with normal pre-pregnancy weight and normal WC. Excessive GWG was found to be associated with an increased risk of LGA [OR 95% CI = 1.74 (1.05–2.89)], but was not significantly related to higher risks of GDM, P-CS and composite outcome [ORs 95% CIs = 0.90 (0.47–1.72), 1.08 (0.77–1.52), and 1.30 (0.94–1.79), respectively]. In terms of the joint effect of maternal pregestational BMI and WC on obesity-related composite outcome, mothers with both pre-pregnancy overweight and central adiposity had the highest risk of composite outcome [OR 95% CI = 3.96 (2.40–6.54)], compared with mothers without pre-pregnancy overweight or central adiposity.

**Conclusions:**

The results of this study suggest that maternal pre-pregnancy overweight/obesity and central adiposity may contribute to multiple obesity-related adverse pregnancy outcomes, excessive weight gain during pregnancy is associated with an increased risk of LGA. Healthcare providers should carry out health education, and guide women to keep an ideal BMI and WC prior to pregnancy and help them gain optimal weight during pregnancy based on their pre-pregnancy BMI and WC.

## Introduction

The universal two-child policy, which is expected to result in a baby boom in China in the near future, has come into effect since 1st January 2016. Consequently, more attention has been attracted regarding how to improve pregnancy outcomes. In recent decades, the prevalence of overweight/obesity among women of childbearing age has been increasing. Among women aged 18–44 years in China, the prevalence of overweight/obesity increased from 19.9% to 33.5% from 1992 to 2010 [1–2].

On the one hand, there’s a positive correlation between age and obesity rate among women of childbearing age [3], but many older women still choose to have a second child; on the other hand, some women might encounter a second pregnancy soon after the previous delivery, carrying along with some additional weight and abdominal fat mass due to high gestational weight gain and postpartum weight retention [4–5]. As a consequence, the prevalence of obesity among women entering pregnancy is likely to increase in China, which will, in turn, result in an increase in obesity-related adverse pregnancy outcomes.

Previous studies have shown that maternal pre-pregnancy overweight/obesity resulted in poor maternal and/or neonatal outcomes, such as gestational diabetes mellitus (GDM), hypertensive disorders (HID), preeclampsia, indicated preterm birth (PTB), macrosomia, large for gestational age (LGA), caesarean section (CS) and instrumental deliveries [6–10]. The association between gestational weight gain (GWG) and pregnancy outcomes remains controversial. A recent study reported that women with excessive GWG had increased risks of CS and delivery of an LGA infant compared with women with adequate weight gain, but women with inadequate GWG had an increased likelihood of GDM [11]. Another study did not find any significant association between GWG and GDM [12]. However, these studies did not control for maternal waist circumference (WC) prior to pregnancy. As an anthropometric index, the WC has been demonstrated to be more representative of visceral fat and central adiposity, and also a better predictor of obesity-related diseases such as type 2 diabetes and cardiovascular disease [13–14]. But few researches have been conducted to reveal the association between maternal WC before pregnancy and pregnancy outcomes. Our objective was to examine the mutual effect of pre-pregnancy body mass index, waist circumference and gestational weight gain on obesity-related adverse pregnancy outcomes.

## Materials and methods

### Study population

This study was based on an in-progress birth cohort, which included a follow-up of participants for five years. This study was conducted under the approval of Independent Ethics Committee Institute of Clinical Pharmacology, Central South University, Changsha, China. (Project number: CTXY-130041-3-2).

Changsha, which is located in central south China, is the capital city of Hunan Province, with more than 3.5 million residents living in six urban districts. Maternal and children health care records in Changsha are available in electronic form from Community Health Management Information System (CHMIS) of Hunan Province. The complete health care procedures for pregnant women and their children are similar to Wuhan City, and has been described in detail in another publication [15].

We randomly selected three Streets of Kaifu District as our study sites, under the following considerations: 1) Kaifu is the largest district in Changsha. 2) due to the limitations of manpower, material and financial resources, we could not afford to conduct our study on a larger scale. 3) our research group has a long-term cooperation with the Health Bureau of Kaifu District, which can ensure the smooth progress of this study.

**Fig 1:**
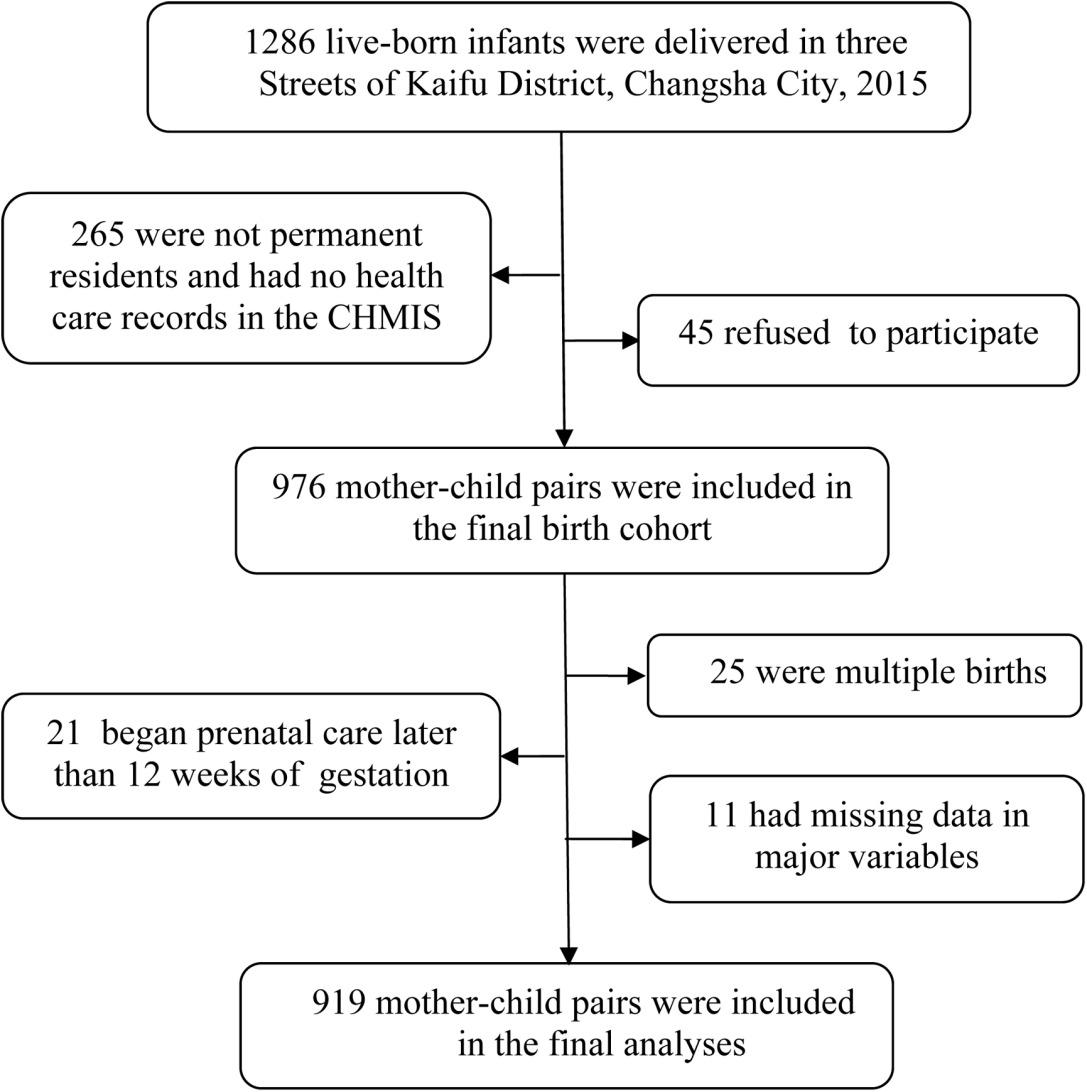
Subject flow diagram. CHMIS, Community Health Management Information System.

From Jan 1, 2015 to Dec 31, 2015, a total of 1,286 infants were born in the three Streets, and the mothers of which that met the following criteria were included in this study: (1) those who delivered live-born babies during this period; (2) those who were permanent residents in the Kaifu District, and whose health care records were registered in the CHMIS; (3) those had no history of mental illnesses or brain diseases, agreed to participate, and provided their written informed consents after being explained about the whole study course. We collected relevant information of mothers, infants and families through a self-administered questionnaire, as well as maternal health manual and CHMIS, within 15 days after deliveries. Eventually, 976 eligible mother-child pairs constituted the final birth cohort. In this analysis, we excluded mothers with multiple births, mothers who initiated antenatal care later than 12 weeks of gestation, and those with missing data in major variables, leaving 919 mother-child pairs for the final analysis (Fig 1. Subject flow diagram.).

### Examinations

Maternal height and weight were measured by a platform scale (RGZ-120-RT, Wuxi Weighing Apparatus Co., China) with participants wearing light clothing and no shoes. Pre-pregnancy BMI was calculated as weight (kg)/height^2^ (m^2^) using data from the records of the first prenatal care visit. According to the Working Group on Obesity in China, BMI was categorized into four levels: < 18.5 kg/m^2^ (underweight), 18.5–23.9 kg/m^2^ (normal weight), 24–27.9 kg/m^2^ (overweight), ≥ 28 kg/m^2^ (obese) [16–17]. Maternal pre-pregnancy WC was measured at the midpoint between the lowest rib and the iliac crest in the standing position with minimal expiration using a standard non-stretch tape at the first prenatal care, and was classified in line with Chinese standard: < 80 cm for non-central adiposity and ≥ 80 cm for central adiposity [18]. GWG was defined as the difference in weight (kg) between maternal weight at delivery and maternal weight at the first prenatal care. Since no official GWG recommendations exist in China, GWG was categorized into three groups based on the Chinese pre-pregnancy BMI classification and the Institute of Medicine (IOM) (2009) recommendations: inadequate (below the recommendations), adequate (within the recommendations), and excessive (above the recommendations). For underweight, normal weight, overweight, and obese women, the IOM-recommended GWG are 12.5–18 kg, 11.5–16 kg, 7–11.5kg and 5–9 kg, respectively [19].

The primary outcomes of interest included: (1) gestational diabetes mellitus (GDM): one or more positive outcomes in 2-hour 75g oral glucose tolerance test (OGTT) between 24 and 28 weeks’ gestation [fasting ≥ 5.1mmol/L (92 mg/dl), 1hour ≥ 10.0 mmol/L(180 mg/dl), 2hour ≥ 8.5 mmol/L (153 mg/dl)]; (2) primary cesarean section (P-CS): first-time cesarean section due to failure to progress, abnormal fetal position or fetal distress, macrosomia, etc., but excluding repetitive CSs; (3) large for gestational age (LGA): birth weight above the 90^th^ percentile, according to Chinese gender-specific birth weight reference curves; (4) composite outcome: one or more of the above adverse pregnancy outcomes.

### Statistical analyses

Continuous variables were described using mean ± standard deviation, while categorical variables using percentage. One-way ANOVA, LSD post hoc test, chi-square test, and partitions of χ^2^ method were used to compare the general characteristics and primary outcomes of mothers and children based on different categories of maternal pre-pregnancy BMI, pre-pregnancy WC and GWG. A scatter plot with a regression line (fitted using least square method) was drawn to visualize the relationship between maternal pre-pregnancy BMI and pre-pregnancy WC. Univariate logistic regression models were used to estimate crude odds ratios (ORs) and the corresponding 95% confidence intervals (CI). Multivariate logistic regression models controlling for the same potential covariates that have shown some evidence for associations with obesity-related pregnancy outcomes in previous studies, including Street, maternal age, maternal education, active or passive smoking, alcohol consumption, family income, parity, paternal age, paternal education, paternal BMI, infant gender and gestational age, were fitted to estimate adjusted ORs and 95% CI. All statistical analyses were performed using SPSS version 20 (IBM, New York, USA).

## Results

Table 1 shows some descriptive statistics and primary obesity-related pregnancy outcomes of mothers and children conditioned on maternal pre-pregnancy BMI, pre-pregnancy WC and GWG categories. Among the 919 mothers, 170 (18.5%) were underweight prior to pregnancy, while 141 (15.3%) were overweight and obese. In terms of weight gain during pregnancy, 171 (18.6%) had insufficient weight gain and 384 (41.8%) had excessive weight gain. The prevalence of central adiposity before pregnancy, according to waist circumference, was 18.3%.

**Table 1:** Characteristics of 919 mother-child pairs according to maternal pre-pregnancy body mass index(BMI), pre-pregnancy waist circumference (WC) and gestational weight gain (GWG) categories in Changsha, China.

		Pre-pregnancy BMI (kg/m^2^)				GWG (kg)				Pre-pregnancy WC (cm)		
Category	All	<18.5	18.5~	≥24	P	Inadequate	Adequate	Excessive	P	<80	≥80	P
No. of subjects	919	170	608	141		171	364	384		751	168	
**Maternal characteristics**												
Age(years)	29.9(3.9)	29.0(3.6)	29.9(3.9)*	30.6(4.1)*	0.002	30.0(4.6)	30.0(3.6)	29.7(3.8)	0.615	29.7(3.9)	30.7(4.1)	0.002
Education(%)					0.398				0.072			0.910
Junior high school and under	3.6	4.1	3.6	2.8		4.7	2.5	4.2		3.6	3.6	
Senior high school	12.8	12.9	11.5	18.4		16.4	9.9	14.1		12.8	13.1	
College	76.0	73.5	77.5	72.3		68.4	80.7	74.7		76.3	74.4	
Graduate and above	7.6	9.5	7.4	6.5		10.5	6.9	7.0		7.3	8.9	
Family income per capita(yuan/ month)(%)					0.251				0.003			0.862
≤2000	3.3	1.2	3.5	5.0		0.6	4.4	3.4^@^		3.1	4.2	
2001–5000	52.9	57.6	50.8	56.0		53.2	50.0	55.5		53.1	51.8	
5001–10000	39.7	36.5	41.3	36.9		41.5	44.0	34.9		39.5	40.5	
>10000	4.1	4.7	4.4	2.1		4.7	1.6	6.2		4.3	3.6	
Pre-pregnancy BMI(kg/m^2^)	21.2(2.9)	17.7(0.6)	21.0(1.5)*	26.5(2.2)*#	<0.001	20.6(2.2)	20.9(2.7)	21.9(3.4)^&^^@^	<0.001	20.4(2.3)	24.8(3.1)	<0.001
GWG(kg)	14.7(4.2)	15.3(3.9)	14.8(4.1)	13.5(4.7)*#	0.001	9.5(1.9)	13.4(2.0)^&^	18.1(3.4)^&^^@^	<0.001	14.6(4.0)	15.1(5.0)	0.143
Pre-pregnancy WC(cm)	75.7(5.3)	71.4(3.2)	75.4(4.1)*	82.3(5.7)*#	<0.001	73.4(4.1)	74.9(4.4)^&^	77.5(5.9)^&^^@^	<0.001	73.8(3.4)	84.0(4.0)	<0.001
Primiparous(%)	70.4	80.6	68.9*	64.5*	0.003	63.2	70.1	74.0^&^	0.036	71.7	67.3	0.324
Active or passive smoking during pregnancy(%)	11.1	8.5	10.5	16.5	0.062	6.1	8.3	15.9^&^^@^	<0.001	10.4	14.2	0.159
Alcohol consumption(%)	5.9	7.1	6.1	3.5	0.386	2.9	5.8	7.3	0.130	6.2	4.8	0.481
GDM(%)	6.2	2.4	5.6	13.5*#	<0.001	6.4	5.5	6.8	0.762	4.5	13.7	<0.001
P-CS(%)	30.5	20.6	29.6	46.1*#	<0.001	25.7	29.1	33.9	0.122	27.0	45.8	<0.001
**Child characteristics**												
Sex(men,%)	52.9	54.7	52.8	51.1	0.812	51.5	52.2	54.2	0.794	53.5	50.0	0.408
Birth weight(kg)	3.36(0.4)	3.21(0.4)	3.37(0.4)*	3.53(0.5)*#	<0.001	3.18(0.4)	3.36(0.4)^&^	3.45(0.4)^&^^@^	<0.001	3.33(0.4)	3.51(0.5)	<0.001
Birth recumbent length(cm)	50.0(0.9)	49.8(0.8)	50.0(0.8)	50.1(1.2)*	0.028	49.8(1.1)	50.0(0.8)^&^	50.0(0.9) ^&^	0.002	50.0(0.8)	50.0(1.4)	0.975
Gestational weeks of birth(wks)	39.0(1.4)	39.0(1.2)	39.1(1.4)	38.9(1.7)	0.702	38.7(1.6)	39.0(1.4)^&^	39.2(1.2)^&^^@^	<0.001	39.1(1.3)	38.9(1.9)	0.191
LGA	10.4	3.0	9.0*	25.5*#	<0.001	4.1	8.0	15.6^&^^@^	<0.001	7.5	23.8	<0.001

Date are means (SD) or percentage.

BMI, body mass index; GWG, gestational weight gain; WC, waist circumference; GDM, gestational diabetes mellitus; P-CS, primary cesarean section; LGA, large for gestational age.

* Compared with groups of pre-pregnancy BMI < 18.5, the difference is statistically significant

# Compared with groups of pre-pregnancy BMI 18.5–23.9, the difference is statistically significant

^&^ Compared with groups of Inadequate GWG, the difference is statistically significant

^@^ Compared with groups of Adequate GWG, the difference is statistically significant.

**Fig 2:**
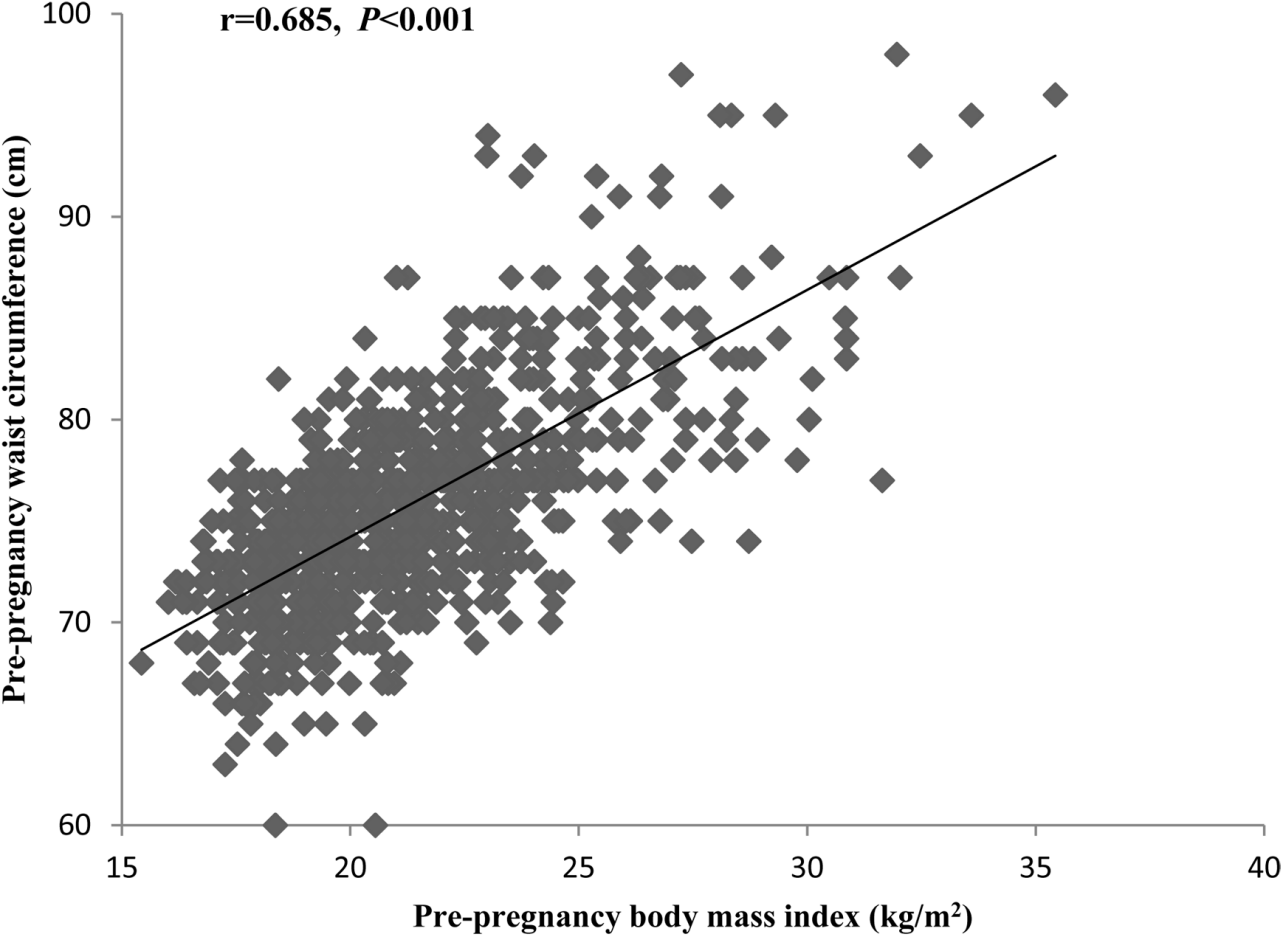
Correlation between maternal pre-pregnancy body mass index (BMI) and pre-pregnancy waist circumference (WC).

A correlation analysis showed a moderately intense and significant correlation between pre-pregnancy BMI and WC (Fig 2. Correlation between maternal pre-pregnancy body mass index (BMI) and pre-pregnancy waist circumference (WC).) (r = 0.685, P < 0.001). Compared with mothers who had normal pregestational weight, mothers who were overweight or obese had a lower GWG and a higher pre-pregnancy WC, and mothers who were underweight were younger, had a lower pre-pregnancy WC and were more often primiparous. Mothers with excessive GWG had a higher pre-pregnancy BMI, a higher pre-pregnancy WC, and were more likely to get involved with active or passive smoking during pregnancy compared with mothers with adequate GWG. Mothers with central adiposity before pregnancy were older, had a higher pre-pregnancy BMI compared with mothers without central adiposity. Maternal pre-pregnancy BMI, WC and GWG were positively related to the birth weight of neonate (Table 1).

The odds ratios (ORs) of obesity-related adverse pregnancy outcomes by pre-pregnancy BMI, WC and GWG were listed in Table 2. After controlling for confounders (Street, maternal age, maternal education, smoking or passive smoking, alcohol consumption, family income, parity, paternal age, paternal education, paternal BMI, infant gender, gestational age), mothers with pre-pregnancy overweight/obesity, central adiposity or excessive GWG all showed an increased risk of LGA [ORs 95% CIs = 1.93 (1.07–3.50), 2.14 (1.21–3.75), and 1.74 (1.05–2.89), respectively] compared with mothers with normal pre-gestational weight, normal waist circumference, and adequate GWG. The elevated risks of developing GDM, P-CS and composite outcome were significantly associated with pre-pregnancy overweight/obesity [ORs 95% CIs = 2.19 (1.02–4.76), 1.66 (1.05–2.65), and 1.82 (1.15–2.87), respectively], and central adiposity [ORs 95% CIs = 2.26 (1.11–4.60), 1.71 (1.11–2.63), and 1.98 (1.30–3.01), respectively]. However, they were not significantly associated with excessive GWG [ORs 95% CIs = 0.90 (0.47–1.72), 1.08 (0.77–1.52), and 1.30 (0.94–1.79), respectively]. Moreover, we also found that pre-pregnancy underweight was significantly associated with lower risks of LGA [OR 95% CI = 0.37 (0.14–0.96)], P-CS [OR 95% CI = 0.64 (0.42–0.98)], as well as composite outcome [OR 95% CI = 0.57 (0.38–0.85)].

**Table 2:** Odd ratios (95% CIs) for obesity-related adverse pregnancy outcomes by pre-pregnancy BMI, WC and GWG.

Category	Pre-pregnancy BMI(kg/m^2^)			GWG(kg)			Pre-pregnancy WC(cm)	
	<18.5	18.5~	≥24	Inadequate	Adequate	Excessive	<80	≥80
**LGA(n = 96)**								
Crude OR(95%CI)	0.31(0.12–0.77)	1	3.44(2.16–5.51)	0.49(0.21–1.15)	1	2.14(1.34–3.42)	1	3.87(2.48–6.06)
Adjusted OR(95%CI)*	0.37(0.14–0.96)	1	1.93(1.07–3.50)	0.63(0.26–1.52)	1	1.74(1.05–2.89)	1	2.14(1.21–3.75)
**GDM(n = 57)**								
Crude OR(95%CI)	0.41(0.14–1.16)	1	2.63(1.45–4.76)	1.18(0.55–2.53)	1	1.25(0.69–2.28)	1	3.35(1.91–5.85)
Adjusted OR(95%CI) *	0.42(0.14–1.24)	1	2.19(1.02–4.76)	1.66(0.73–3.78)	1	0.90(0.47–1.72)	1	2.26(1.11–4.60)
**P-CS(n = 280)**								
Crude OR(95%CI)	0.62(0.41–0.93)	1	2.03(1.40–2.96)	0.84(0.56–1.27)	1	1.25(0.91–1.70)	1	2.28(1.62–3.22)
Adjusted OR(95%CI) *	0.64(0.42–0.98)	1	1.66(1.05–2.65)	0.96(0.61–1.49)	1	1.08(0.77–1.52)	1	1.71(1.11–2.63)
**Composite outcome(n = 366)**								
Crude OR(95%CI)	0.52(0.35–0.76)	1	2.62(1.79–3.82)	0.75(0.51–1.11)	1	1.51(1.13–2.03)	1	3.03(2.15–4.29)
Adjusted OR(95%CI) *	0.57(0.38–0.85)	1	1.82(1.15–2.87)	0.86(0.57–1.31)	1	1.30(0.94–1.79)	1	1.98(1.30–3.01)

BMI, body mass index; GWG, gestational weight gain; WC, waist circumference; GDM, gestational diabetes mellitus; P-CS, primary cesarean section; LGA, large for gestational age.

* Adjusted for Street, maternal age, maternal education, smoking or passive smoking, alcohol consumption, family income, parity, paternal age, paternal education, paternal BMI, infant gender, gestational age.

**Fig 3:**
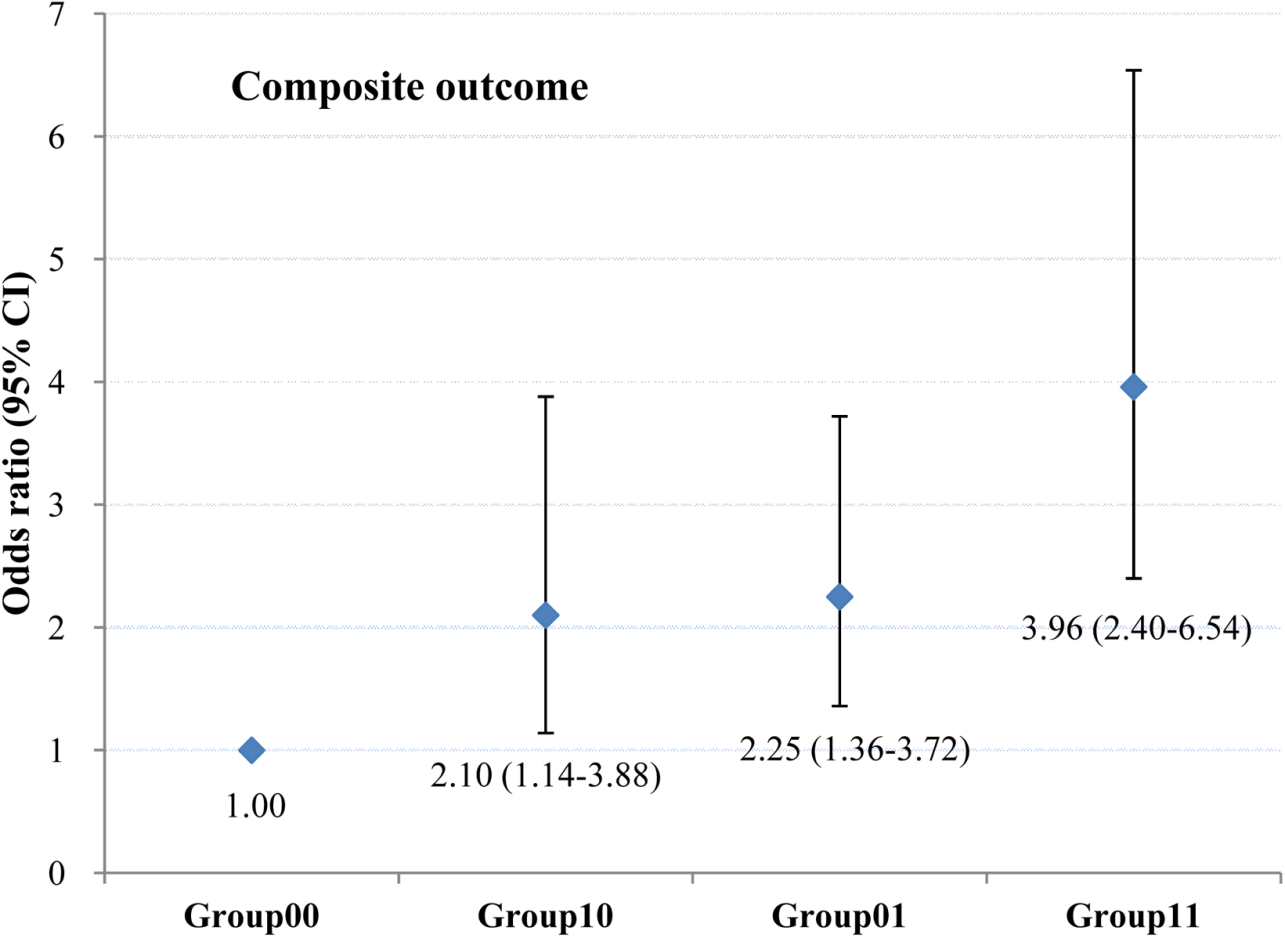
Odds ratios (95% CIs) for obesity-related composite outcome by joint effect of pre-pregnancy body mass index (BMI) and waist circumstance (WC). Group 00: Pre-pregnancy BMI < 24 kg/m^2^ and Pre-pregnancy WC < 80 cm; Group 10: Pre-pregnancy BMI ≥ 24 kg/m^2^ and Pre-pregnancy WC < 80 cm; Group 01: Pre-pregnancy BMI < 24 kg/m^2^ and Pre-pregnancy WC ≥ 80 cm; Group 11: Pre-pregnancy BMI ≥ 24 kg/m^2^ and Pre-pregnancy WC ≥ 80 cm.

We further evaluated the joint effect of maternal pregestational BMI and WC on obesity-related composite outcome by classifying the mothers into four groups: 1) Group00: mothers without pre-pregnancy overweight or central adiposity (BMI < 24 kg/m^2^ and WC < 80 cm); 2) Group10: mothers with pre-pregnancy overweight but without central adiposity (BMI ≥ 24 kg/m^2^ and WC < 80 cm); 3) Group01: mothers with pre-pregnancy central adiposity but without pre-pregnancy overweight (BMI < 24 kg/m^2^ and WC ≥ 80 cm); 4) Group11: mothers with both pre-pregnancy overweight and central adiposity (BMI ≥ 24 kg/m^2^ and WC ≥ 80 cm). Compared with mothers without pre-pregnancy overweight and central adiposity, mothers with both pre-pregnancy overweight and central adiposity had the highest risk of composite outcome [OR 95% CI = 3.96 (2.40–6.54)], mothers without pre-pregnancy overweight but with central adiposity had a higher risk of composite outcome [OR 95% CI = 2.25 (1.36–3.72)] (Fig 3. Odds ratios (95% CIs) for obesity-related composite outcome by joint effect of pre-pregnancy body mass index (BMI) and waist circumstance (WC).).

## Discussion

In this birth cohort study, we examined the mutual effect of pre-pregnancy body mass index, waist circumference and gestational weight gain on obesity-related adverse pregnancy outcomes. In our cohort of 919 pregnant women, the prevalence of pre-pregnancy overweight/obesity was 15.5%, which is a bit higher than the previously reported prevalence in Chinese women (10.4%) and Korean women (9.4%), mainly because the BMI categories they used were WHO classification criteria, while we used Chinese adult BMI classification criteria that were lower than the former for overweight and obese [11,20]. However, the prevalence of pre-pregnancy overweight/obesity in our cohort is significantly lower than the prevalence in a Ghanaian cohort study (48.2%) and an Australian longitudinal cohort study (54.7%) [21–22]. 41.8% of pregnant women in this population were above the recommended GWG ranges by IOM guidelines(2009). Recently, a cohort study in Wuhan, China reported that 57.5% of pregnant women had excessive GWG [15]. While another prospective multicentre study in Italy reported 29.5% of women gained excessive weight than IOM-recommended ranges [23]. Therefore, the IOM GWG guidelines often used in Western populations may be not appropriate for Chinese populations. However, there’s not an official GWG recommendation based on Chinese BMI cut-off points for Chinese women. 18.3% of women in our study had central adiposity prior to pregnancy, which is lower than the previously reported percentage (25.7%) in developed countries [24]. In general, the status of pre-pregnancy weight and waist circumference in our cohort was less than ideal.

The results of this study suggested that both maternal pre-pregnancy overweight/obesity and pre-pregnancy central adiposity were associated with increased risks of gestational diabetes mellitus, primary cesarean section, large for gestational age, as well as composite outcome, meanwhile, excessive weight gain during pregnancy was associated with an increased risk of large for gestational age.

The relationships between maternal pre-pregnancy BMI and GDM, LGA and P-CS have been reported in many studies [6–7,11–12]. A previous research involving 33,973 pregnant women in Tianjin, China revealed an important role of high maternal pre-pregnancy BMI in the development of GDM [12]. Recently, a systematic review concluded that high BMI before pregnancy increased the risk of LGA and subsequent offspring overweight/obesity compared with women with normal BMI before pregnancy [6]. Zilberlicht et al, found that high pre-pregnancy BMI independently contributed to adverse pregnancy outcomes such as LGA and P-CS in a retrospective study of 8,595 Israeli women [7]. The present study confirmed those findings, we found that mothers with pre-pregnancy overweight or obesity had a 2.19 fold the risk of developing GDM, 1.93 fold risk of delivering an LGA infant, and 1.66 fold risk of experiencing P-CS, as well as 1.82 fold risk of developing composite outcome in comparison with those having normal pre-pregnancy BMI. Furthermore, the present study also suggested that maternal pre-pregnancy underweight was significantly associated with lower risks of LGA, P-CS, as well as composite outcome, However, it may be associated with increased risks of other adverse pregnancy outcomes, such as small for gestational age (SGA) and spontaneous preterm birth [6,25], which was not studied in this article.

Consistent with previous studies [7, 11–12], this study found that women with excessive GWG had an increased risk of delivering an LGA infant compared with women who had adequate GWG. Li et al [26] reported that maternal excessive GWG might have a greater effect on the offspring’s overweight, and might contribute to the infants’ and children’s overweight epidemic. Meanwhile, we did not find any significant association of GWG with GDM or P-CS, which was different from some studies [7,11,27–28], but similar to other studies [12,29–30]. A previous study reported that the rate of gestational weight gain (RGWG) up to mid pregnancy was significantly associated with a higher risk of developing GDM (OR, 2.22; 95% CI, 1.43–3.47), but RGWG at late pregnancy had a lower risk of GDM (OR, 0.29; 95% CI, 0.14–0.33) [20]. That was probably because after being diagnosed with GDM, women would take some actions, such as diet modification, physical activity and/or insulin therapy, to control the weight gain in later pregnancy. Hence, GWG during the entire gestation period may be not a good predictor of GDM. The association of GWG with cesarean delivery also remains controversial. Some researches suggested women with excessive GWG were more likely to experience P-CS [7,20], while other researches, including ours, showed that GWG did not have a statistically significant influence on cesarean delivery after adjusted for potential confounders [29–30]. Moreover, after controlling the confounders, nor did we find any association between GWG and composite outcome. However, more samples and new Chinese BMI-specific GWG recommendations are needed to reveal the association between GWG and obesity-related adverse pregnancy outcomes.

There are few previous studies about the influence of maternal central adiposity on pregnancy outcomes [31–33]. Suresh et al, in a stratified cohort study involving 1,200 pregnant women in western Sydney, Australia, noted that abdominal fat mass was a stronger risk factor for GDM, CS and LGA than pre-pregnancy BMI [32]. Similar to this study, our study found that mothers with pre-pregnancy central adiposity had a 2.26 fold risk of developing GDM, 2.14 fold risk of delivering an LGA infant, and 1.71 fold risk of experiencing P-CS, as well as 1.98 fold risk of developing composite outcome compared with those mothers without pre-pregnancy central adiposity. A meta-regression analysis of prospective studies has suggested that central adiposity was related to the presence of visceral adipose tissue, which contributed to the development of insulin resistance [34], and these may be more obvious during pregnancy. Another cohort study of 3,083 primiparous women in the United Kingdom, reported that women with waist to hip ratios in the third and fourth quartiles of the study population were associated with an increased risk of infant LGA, defined as birth weight ≥ 95^th^ percentile of the study population [33]. But McDonnold et al, in a cohort of 2,276 low-risk nulliparous women in the USA, did not find any association of central adiposity with increased risks of infant LGA, the author argued that this was possibly due to the low-risk nature of their cohort [35]. Two reasons may help to explain in part that women with pre-pregnancy central adiposity were more likely to experience P-CS. The accumulation of adipose tissue within the abdomen and pelvis could distort the pelvic outlet, which would influence the descending of the fetal head. In addition, the predisposition of women with pre-pregnancy central adiposity to have larger size babies may also contribute the increased risk of cesarean section [36]. These all indicated that maternal pre-pregnancy WC played an important role in the predictor of obesity-related adverse pregnancy outcomes.

Our study also evaluated the joint effect of maternal pregestational BMI and WC on obesity-related composite outcome. We found that mothers with both pre-pregnancy overweight and central adiposity had the highest risk of composite outcome, while mothers without pre-pregnancy overweight but with central adiposity had a higher risk of composite outcome in comparison with mothers without pre-pregnancy overweight or central adiposity. Our study indicated that maternal pre-pregnancy WC played a more important part than pre-pregnancy BMI in obesity-related adverse pregnancy outcomes. Therefore, in the future, healthcare providers should pay more attention to how to encourage women with central adiposity to get healthy pregestational waist circumference.

This study has several strengths and limitations. To our knowledge, this is the first study to examine the mutual effect of pre-pregnancy body mass index, waist circumference and gestational weight gain on obesity-related adverse pregnancy outcomes. Additionally, this is a community-based birth cohort study, we collected data by a self-administered questionnaire, not just by excerpting from maternal health manual and CHMIS, which enabled adjustment for multiple potential confounders. However, this study also has some limitations. A notable one is that the sample size was relatively small, and the population only comprised of women from the urban districts of Changsha, so the conclusions may not be generalizable to all women. Secondly, anthropometric data were retrieved from the records of the first prenatal care visit within 12 weeks of gestation, which may lead to some information bias and misclassification. Another limitation is that the IOM GWG categories we used were based on western populations, which may be not appropriate for Chinese populations. Therefore, further high-quality, large-scale mother-infant cohort studies are needed to develop Chinese BMI-specific and WC-specific GWG recommendations. Lastly, we only examined the mutual effect of pre-pregnancy BMI, WC and GWG on short-term pregnancy outcomes, but long-term effects such as subsequent maternal complications and postpartum weight retention, as well as offspring’s growth and development were not examined. However, this birth cohort study is an in-progress project, and hopefully we will obtain relevant information in the near future.

## Conclusions

To conclude, our study suggests that maternal pre-pregnancy overweight/obesity and central adiposity may contribute to multiple obesity-related adverse pregnancy outcomes, excessive weight gain during pregnancy is associated with an increased risk of LGA. To improve pregnancy outcomes, the following interventions are needed. Firstly, healthcare providers should carry out health education to raise the public awareness of the risks of maternal pre-pregnancy obesity, central adiposity and weight gain during pregnancy on mothers and their infants. Secondly, women should be guided to keep an ideal BMI and WC prior to pregnancy, to gain optimal weight during pregnancy and to return to a healthy BMI and WC within a reasonable amount of time before the second pregnancy. Last but not least, offering women of childbearing age clinical guidelines on nutrition, diet, physical activity, weight and waist circumference management, as well as emphasizing the importance of self-monitoring.

## Supporting information

S1 File Chart data. (XLSX)
